# Radiological Finding of Crazy-Paving Pattern in COVID-19 Pneumonia

**DOI:** 10.7759/cureus.26107

**Published:** 2022-06-20

**Authors:** Wahab A Gbadamosi, Brandon Hanai, Paul Kim, Tyson Anthony, Zenaida Rivera

**Affiliations:** 1 Diagnostic Radiology, Medical Center of Trinity, Trinity, USA; 2 Medicine, American University of Antigua, Coolidge, ATG

**Keywords:** thorax radiology, acute hypoxic respiratory failure, sars-cov-2 (severe acute respiratory syndrome coronavirus -2), crazy paving pattern, covid-19 pneumonia

## Abstract

The recent global pandemic of coronavirus disease 2019 (COVID-19) has brought many radiographic findings in other respiratory disease processes. One of these radiological findings is crazy paving. This paper discusses crazy paving in a 75-year-old female with dyspnea, nonproductive cough, pleuritic chest pain, and a polymerase chain reaction (PCR) positive test for COVID-19 infection. Chest CT showed ground-glass opacities and interlobular septal thickening consistent with a crazy-paving appearance. As part of the common CT findings of patients with active COVID-19 infection, crazy paving should prompt the interpreting radiologist to consider COVID-19 pneumonia as part of the differential.

## Introduction

Coronavirus disease 2019 (COVID-19) was declared a pandemic by the World Health Organization on March 11, 2020, and has affected the medical community worldwide, leading to massive mortalities [1]. COVID-19 is caused by the severe acute respiratory syndrome coronavirus 2 (SARS-CoV-2) virus. Diagnosis is based on clinical symptoms and laboratory tests. However, given the multiple complications associated with COVID-19, radiological imaging is often obtained to exclude these complications. Radiological imaging of COVID-19 shows similarities with other acute or chronic pulmonary etiologies on contrast tomography [1-3]. One of these chest CT imaging findings is crazy paving which was initially a pathognomonic sign in patients diagnosed with pulmonary alveolar proteinosis [4,5]. Therefore, this case report aims to describe a radiological CT finding of crazy paving in COVID-19 pneumonia.

## Case presentation

We present a case of a 75-year-old female patient who presented to the hospital for acute worsening dyspnea that was progressively worsening. A week before hospital presentation, the patient reported having mild chest discomfort that progressively worsened into pleuritic chest pain, nonproductive cough, malaise, generalized weakness, and nausea. The patient did not endorse diarrhea, fever, diaphoresis, dizziness, palpitation, or recent travel. The past medical and surgical history was non-contributory. There were no known allergies; the patient was not on any home medication and denied tobacco, alcohol, or recreational drug use. Family history was significant for diabetes and cardiovascular disease.

On physical examination, the patient’s temperature was 39.5℃, heart rate was 97 beats per minute, blood pressure was 115/59 mmHg, respiratory rate was 18 beats per minute, and oxygen saturation ranged between 80%-93% on 4 liters of oxygen via nasal cannula. The patient had respiratory distress while talking but was alert and oriented to self, time, place, and situation. The pupils were anicteric, round, and reactive to light. Mucosal membranes were dry, the neck was supple, and there was no palpable cervical lymphadenopathy. The lungs exam was significant for poor inspiratory effort and bilateral crackles. The cardiac exam was normal S1/S2 without murmur, rubs, or gallops. The abdominal exam was soft, non-distended, with normal bowel sounds and no guarding or costovertebral region tenderness. Neurological exam shows intact cranial nerve 2-12, no sensory, motor, or reflex deficits. The remainder of the exam was non-contributory.

Laboratory findings were significant for leukocytosis (22,000/uL), thrombocytosis (524,000uL) hemoglobin (Hgb) 16.3g/dl, hematocrit 49.8%. polymerase chain reaction (PCR) for SARS-COV-2 was positive. D-dimer and C-reactive protein (CRP) was elevated at 1.22mg/L (0.19-0.50mg/L) and 6.34mg/dl. Respiratory alkalosis on arterial blood gas showed potential of hydrogen (pH)/partial pressure of carbon dioxide (PCO2)/bicarbonate (HCO3)/O2 of 7.5/43/33.6/83. The blood culture was negative. The comprehensive metabolic panel (CMP) and urinalysis were unremarkable.

**Figure 1:**
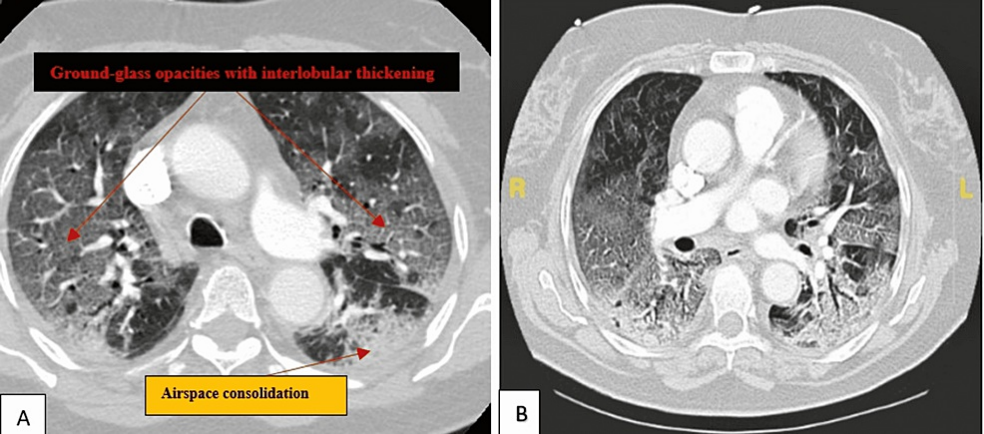
Image A and B shows axial view of the chest CT lung window demonstrating ground-glass opacities with interlobular septal thickening and bibasilar airspace opacities.

The electrocardiogram (ECG) showed sinus tachycardia and no ST/T wave changes. Chest frontal radiograph demonstrates diffuse bilateral pulmonary airspace and interstitial opacities. CT angiography (CTA) of the chest was negative for pulmonary embolism but significant for bilateral ground-glass opacities with interlobular septal thickening (Figure 1). This imaging finding was consistent with crazing paving radiographic findings in the setting of COVID-19 pneumonia.

Specialists in pulmonary and infectious disease were consulted throughout the hospital course. The patient was treated appropriately with medical therapy for acute hypoxic respiratory failure.

## Discussion

Crazy paving* *is often used in imaging because its appearance looks like a path made with broken pieces of concrete [1]. This term was initially a pathognomonic sign in patients diagnosed with pulmonary alveolar proteinosis, which is a diffuse lung disease characterized by the accumulation of lipoproteinaceous material within the distal airspaces due to surfactant production or metabolism [4-5]. This pattern has been described in various acute and chronic pulmonary etiologies. The differential diagnosis for crazy paving includes Pneumocystis jirovecii pneumonia, mucinous bronchioloalveolar carcinoma, sarcoidosis, organizing pneumonia, nonspecific interstitial pneumonia, lipoid pneumonia, adult respiratory distress syndrome, and pulmonary hemorrhage syndrome [2,3,6]. Given the novelty of COVID-19 in the medical community and the imaging obtained based on our patient, it is suggested COVID-19 pneumonia be added to the differential for patients with the radiographic findings of crazy paving on CT chest.

The pathophysiology of crazy paving in COVID-19 is similar to the Middle East respiratory syndrome and severe acute respiratory syndrome, which involves the pulmonary alveolar airspace and interstitial networks [7]. It starts with the host cell entry of the virus into the alveolar epithelial cells after inhalation from the upper respiratory pathway. The viral spike protein attaches to angiotensin-converting enzyme II and transmembrane serine protease 2 [7,8]. Once the host macrophage recognizes this antigen, a downstream cascade occurs, leading to the excessive activation of pro-inflammatory cytokines, referred to as a storm. This cytokine storm leads to a hyperinflammatory response, which in turn causes acute lung injury and respiratory failure [8]. This lung injury is manifested on imaging as thickening of the pulmonary interlobular septal due to ongoing proteinous fluid accumulation, which eventually fills the airspace and manifests as ground-glass opacities in the acute setting. These pathophysiology findings correlate with our patient imaging findings.

The radiological chest CT features of crazy paving in COVID-19 pneumonia include peripheral ground-glass opacities with thickened interlobular septa, which define crazy paving and airspace consolidation [9]. Our patient's chest CT showed these radiological findings of crazy paving, as shown in Figure 1.

## Conclusions

A crazy-paving pattern is a CT manifestation of many diverse medical entities in radiological images. Therefore, in patients with acute dyspnea and clinical signs of a viral etiology, along with crazy paving on CT images, it is suggested that COVID-19 pneumonia be included in the differential diagnosis. Ongoing studies on the constellation of CT pulmonary findings, including crazy-paving patterns in patients with active COVID-19 infection, will continue to contribute to clear and concise diagnosis considering other differentials. 
